# An Integrated LC-ESI-MS^n^ and High Resolution LC-ESI-QTOF Approach for the Identification of Phloroglucinols from Nepalese *Hypericum japonicum*

**DOI:** 10.3390/molecules25245937

**Published:** 2020-12-15

**Authors:** Gregorio Peron, Deepak Raj Pant, Shyam Sharan Shrestha, Sangeeta Rajbhandary, Stefano Dall’Acqua

**Affiliations:** 1 Department of Pharmaceutical and Pharmacological Sciences, University of Padova, Via Marzolo 5, 35131 Padova, Italy; 2 Central Department of Botany, Tribhuvan University, Kirtipur 44600, Kathmandu, Nepal; drpant_agbot@yahoo.com (D.R.P.); shyamsharan999@gmail.com (S.S.S.); s.rajbhandary@cdbtu.edu.np (S.R.)

**Keywords:** *Hypericum japonicum*, phloroglucinols, mass spectrometry, dereplication

## Abstract

Phloroglucinols are characteristic constituents of *Hypericum* *japonicum* that are claimed to exert several bioactivities, such as anti-inflammatory, anti-depressant and anti-viral ones. Phloroglucinols are unstable compounds and their synthesis is challenging; thus, isolation from natural sources is still one of the main strategies for obtaining these constituents in purified form. Assessing the presence of phloroglucinols in plant materials can be of interest for compound isolation, and LC-MS approaches afford sensitivity and specificity in this regard. In this work, we combined data from quadrupole-time of flight (QTOF) and ion trap (IT) mass spectrometers in order to assess the presence of the phloroglucinols characteristic of *H. japonicum* and to elucidate their MS fragmentation pathways. The identified compounds present similar structures bearing the 1,3,5-trihydroxybenzene core with different substitutions, which, in constituents at higher MW, is linked to 3′,3′-dimethyl-6′-oxo-phlorisobutyrophenone by a methylene bridge. Differences in MS^2^ spectra of the considered phloroglucinols are useful for compound identification and differentiation, and to perform dereplication studies. Overall, the proposed approach could be useful for the analysis of phloroglucinols in *H. japonicum* and other plant species.

## 1. Introduction

Phloroglucinols are naturally occurring derivatives of 1,3,5-trihydroxy-benzene encountered in several plant species [1]. Acylated phloroglucinols, characteristic constituents of *Hypericum* plants, have been correlated with some of the biological activities of this genus, so they are considered as potential novel therapeutic agents [2,3]. In a recent review published by Bridi and coll. [3], the authors summarized the distributions and biological activities of more than 400 phloroglucinol derivatives isolated from *Hypericum* species, as proof of the growing interest devoted to these bioactive compounds. Most importantly, hypericin and pseudohypericin from *H. perforatum* have been considered as responsible for its anti-depressant activity [4]. Uliginosin B, a dimeric acylphloroglucinol from *H. uliginosum* and *H. brasiliense*, among the others, has shown antinociceptive [5] and antidepressant-like activities in mice [6], antiprotozoal activity against *Trichomonas vaginalis* [7] and antibacterial activity against *Staphylococcus aureus* and *S. epidermidis* [8]. Three prenylated phloroglucinols isolated from the leaves of *H. scruglii* from Sardinia (Italy) have been identified as novel dual HIV-1 inhibitors, and were able to inhibit the HIV-1 replication in cell-based assays [9]. Bioactivity-guided fractionation of *H. empetrifolium* and *H. japonicum* extracts led to the isolation of acylphloroglucinols that were considered the active compounds responsible for the observed in vitro anti-inflammatory and anti-proliferative activities [10]. The same activities have been recently reported for the methanolic extract of *H. japonicum* from Nepal, and it has been supposed that the eleven phloroglucinol derivatives identified among the secondary metabolites could be co-responsible for the biological effects observed in vitro [11].

Even though synthetic approaches to obtaining substituted acylphloroglucinols from 1,3,5-trihydroxy-benzene are challenging [1], their isolation from natural sources is actually the most straightforward strategy to provide purified compounds for pharmacological evaluations and further drug development. However, reference standards for these compounds are difficult to find on the market; hence, there is a need for methods that allow their fast (although tentative) identification on the basis of experimental data, such as the analysis of fragmentation patterns using multiple-level tandem mass spectrometry (MS^n^). The hyphenation of liquid chromatography (LC) with ion trap MS allows one to monitor the multi-stage fragmentation of single components in a complex mixture, without the need for previous isolation [12,13]. Furthermore, the utilization of high-resolution MS (HR-MS), such as quadrupole-time of flight (QTOF) instrumentation, allows one to obtain information about the accurate masses of these components, and to calculate their molecular formulas. Hence, analytical data obtained from integrated LC-MS^n^ and LC-QTOF approaches are sufficient to perform tentative assignation of phloroglucinols directly from natural extracts and without the use of reference standards, allowing one to perform rapid screening of natural sources for their content in these bioactive compounds.

In this study, as a continuation of our previous work on Nepalese *H. japonicum* [11], we report the detailed MS fragmentation of eleven phloroglucinol derivatives previously identified in the methanol extract of the same species. These compounds represent characteristic constituents of *H. japonicum* and could be of potential interest for the development of novel anti-inflammatory and anti-proliferative agents [11,14]. Using multi-stage structural information and accurate *m*/*z* values obtained from an integrated LC-MS^n^ (3D ion trap) and LC-QTOF approach, we evaluated the fragmentation pathways of phloroglucinols directly from the methanol extract of *H. japonicum* (HJME). Overall, the data presented here could represent useful information for the qualitative analysis of phloroglucinols from *H. japonicum* and other plant species, and could help their characterization and identification from crude extracts for the further development of novel therapeutic agents.

## 2. Results and Discussion

**Figure 1:**
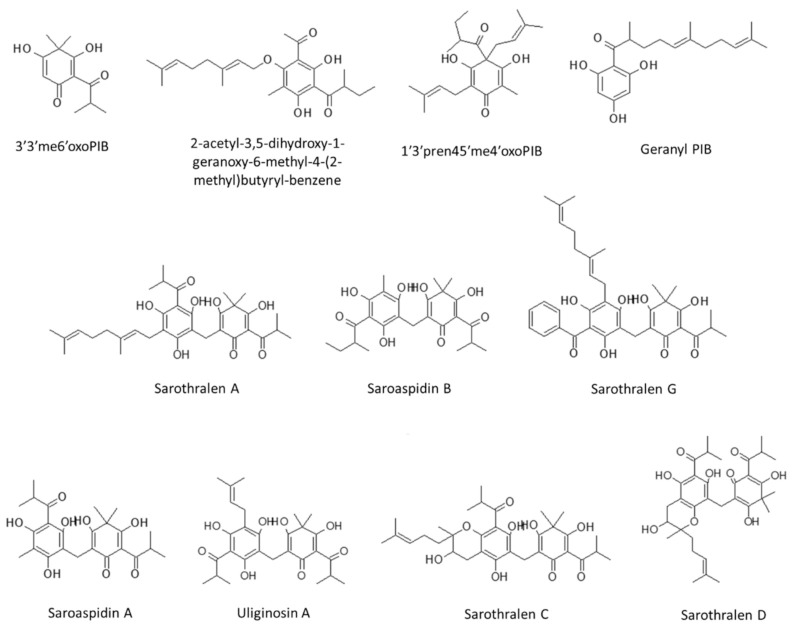
Chemical structures of the phloroglucinol derivatives characterized in HJME. PIB: phlorisobutyrophenone; 3′3′me6′oxoPIB: 3′,3′-dimethyl-6′-oxo-phlorisobutyrophenone; 1′3′pren45′me4′oxoPIB: 1’,3’-diprenyl-4,5’-dimethyl-4’-oxo- phlorisobutyrophenone.

**Figure 2:**
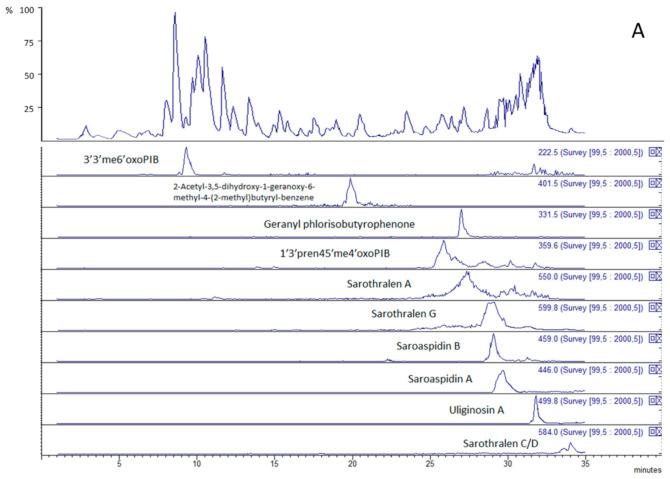
Representative chromatogram obtained from LC-ESI(-) ion trap analysis of *H. japonicum* extract (panel A) and extracted ion chromatograms of the characterized phloroglucinol derivatives. Each chromatographic trace shows the peak(s) associated with a specific phloroglucinol, whose name is indicated. 3′3′me6′oxoPIB: 3′,3′-dimethyl-6′-oxo-phlorisobutyrophenone; 1′3′pren45′me4′oxoPIB: 1’,3’-diprenyl-4,5’-dimethyl-4’-oxo-phlorisobutyrophenone.

The chromatographic method here presented allows the separation of the main phloroglucinol derivatives of HJME in a 35 min-long gradient on a C-18 column. The chemical structures of the analyzed compounds are reported in Figure 1. Representative LC-MS ion trap chromatograms of the whole HJME showing the base peak ion trace and the extracted ion chromatograms of each identified phloroglucinol derivative are reported in Figure 2. As proposed by other authors [15], due to the fact that not all the phloroglucinols have trivial and commonly used names, they will be referred to by abbreviations according to their chemical properties, referring to the basic structure of phlorisobutyrophenone (PIB) and its related derivatives.

The tentative identification of the eleven phloroglucinols from HJME has been performed by comparing the accurate *m*/*z* values obtained by QTOF analysis and the main fragments formed by MS/MS with literature data [15], and they have been already reported in [11]. The aim of the present work was that of going further, by exploring the fragmentation pathways of the single phloroglucinols and determining characteristic fragments that could help with their determination in *H. japonicum* extracts and other plant materials. 

**Figure 3:**
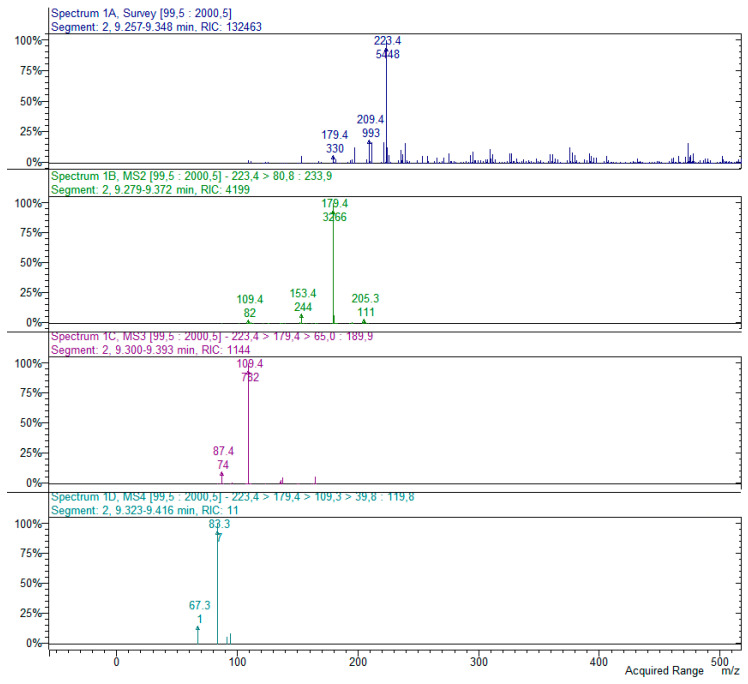
MS^n^ spectra (*n* = 4) of 3′3′me6′oxoPIB, [M-H]^−^ = 223. The spectra show the main fragments formed by the sequential fragmentation of the parent compound (*m*/*z* 223 > 179) and its main products (*m*/*z* 179 > 109; *m*/*z* 109 > 83). The structures of the main fragments are reported in the scheme in Figure 4.

**Figure 4:**
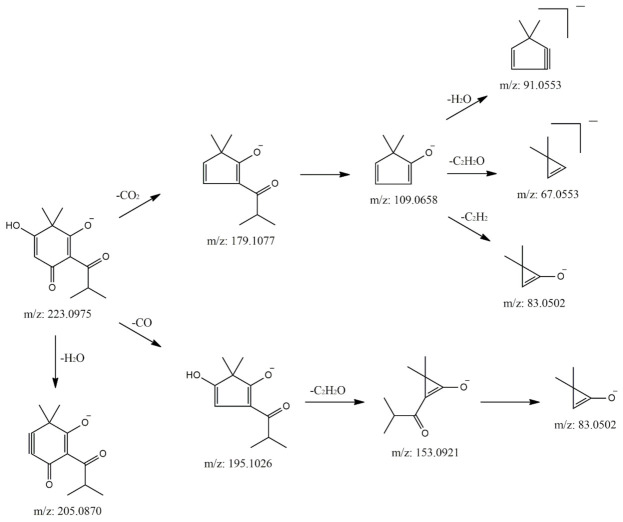
Proposed fragmentation scheme for 3′3′me6′oxoPIB, [M-H]^−^ = 223. The main fragments are shown together with information about their *m*/*z* values. The mass spectra with the peaks assignable to each fragment are reported in Figure 3.

The HR-MS analysis of 3′,3′-dimethyl-6′-oxo-PIB (3′3′me6′oxoPIB) yielded a *m*/*z* value of 223.0961 for its [M-H]^−^ ion (Δ = 4.28 ppm for C_12_H_15_O_4_^−^). The MS2 spectrum in ion trap (Figure 3) shows the characteristic fragment [15] at *m*/*z* 179 (*m*/*z* 179.1064 from HR-MS; Δ = 4.73 ppm for C_11_H_15_O_2_^−^), presumably formed by the loss of CO_2_ (−44 amu) (see the fragmentation scheme in Figure 4). Two other main fragments were observed in the MS2 spectrum, namely, that at *m*/*z* 205 (*m*/*z* 205.0865 from HR-MS; Δ = 5.69 ppm for C_12_H_13_O_3_^−^), formed from the elimination of a molecule of water (−18 amu), and that at *m*/*z* 153 (*m*/*z* 153.0545 from HR-MS; Δ = 4.85 ppm for C_8_H_9_O_3_^−^), formed by the loss of 70 amu, corresponding to C_4_H_7_O (isobutyryl moiety). Another minor fragment at *m*/*z* 195 (*m*/*z* 195.1016 from HR-MS; Δ = 5.97 ppm for C_11_H_15_O_3_^−^) supports the loss of CO (−28 amu) (Figure 4). 

The MS3 fragmentation of the base peak ion at *m*/*z* 179 yielded the formation of a main signal at *m*/*z* 109, due to the loss of the isobutyryl moiety (−70 amu), as observed before. MS4 spectrum generated from the ion at *m*/*z* 109 shows a base peak fragment at *m*/*z* 83, corresponding to loss of C_2_H_2_ (−26 amu) from the ring, and signals at *m*/*z* 91 and 67, corresponding respectively to the loss of water and C_2_H_2_O (−42 amu). Overall, the fragments at *m*/*z* 109 and 83 could be considered as characteristic of 3′3′me6′oxoPIB fragmentation, together with that at *m*/*z* 179, already reported in [15].

The MS2 spectrum generated from the fragmentation of 2-acetyl-3,5-dihydroxy-1-geranoxy-6-methyl-4-(2-methyl)butyryl-benzene (*m*/*z* 401.2313 from HR-MS; Δ = 5.28 ppm for C_24_H_33_O_5_^−^) shows two intense signals at *m*/*z* 221 (*m*/*z* 221.0808 from HR-MS; Δ = 5.27 ppm for C_12_H_13_O_4_^−^) and 177 (*m*/*z* 177.0912 from HR-MS; Δ = 5.39 ppm for C_11_H_13_O_2_^−^) (Figure S1). The first fragment is generated after the breakage of the geranyl chain (−138 amu) from the ether in position 3 of the ring and the acetyl group (−42 amu) (Fragmentation Scheme S1, Supplementary Material). The loss of a molecule of CO_2_ from this structure yields the fragment at *m*/*z* 177, in a step similar to that observed for 3′3′me6′oxoPIB.

The pseudomolecular ion of 1′,3′-diprenyl-4,5′-dimethyl-4′-oxo-PIB (1′3′pren45′me4′oxoPIB) shows an *m*/*z* value of 359.2209 in HR-MS analysis (Δ = 3.84 ppm for C_22_H_31_O_4_^−^). The MS2 spectrum (Figure S2) shows a base peak at *m*/*z* 289 (*m*/*z* 289.1429 from HR-MS; Δ = 7.70 ppm for C_17_H_21_O_4_^−^), formed after the loss of a prenyl group (−70 amu). This can cause the loss of an isobutenyl group (−56 amu) to give a second intense signal at *m*/*z* 233 (*m*/*z* 233.0809 from HR-MS; Δ = 4.55 ppm for C_13_H_13_O_4_^−^), from which the fragment at *m*/*z* 205 (*m*/*z* 205.0855 from HR-MS; Δ = 7.75 ppm for C_12_H_13_O_3_^−^) is formed after the elimination of a CO. The main fragment at *m*/*z* 289 can also undergo the typical elimination of CO_2_ previously observed for the two derivatives described above, leading to the signal at *m*/*z* 245 (*m*/*z* 245.1534 from HR-MS; Δ = 5.62 ppm for C_16_H_21_O_2_^−^) (Fragmentation Scheme S2, Supplementary Material).

The loss of CO from the pseudomolecular ion leads to the fragment at *m*/*z* 331 (*m*/*z* 331.2261 from HR-MS; Δ = 3.84 ppm for C_21_H_31_O_3_^−^), which undergoes the elimination of several methyl radicals (−15 amu each) and C_2_H_2_ groups to form the fragments at *m*/*z* 301 (*m*/*z* 301.1789 from HR-MS; Δ = 4.92 ppm for C_19_H_25_O_3_^−^), 275 (*m*/*z* 275.1634 from HR-MS; Δ = 7.32 ppm for C_17_H_23_O_3_^−^), 260 (*m*/*z* 260.1408 from HR-MS; Δ = 4.07 ppm for C_16_H_20_O_3_^−^), 245 (*m*/*z* 245.1171 from HR-MS; Δ = 5.19 ppm for C_15_H_17_O_3_^−^) and 219 (*m*/*z* 219.1015 from HR-MS; Δ = 5.80 ppm for C_13_H_15_O_3_^−^)—which after the elimination of CO, generates the fragment at *m*/*z* 191 (*m*/*z* 191.1066 from HR-MS; Δ = 6.66 ppm for C_12_H_15_O_2_^−^) (Fragmentation Scheme S2, Supplementary Material).

The fragmentation of geranyl-PIB (*m*/*z* 331.1897 from HR-MS; Δ = 3.84 ppm for C_20_H_27_O_4_^−^) yields the base peak fragment at *m*/*z* 287 (*m*/*z* 287.1272 from HR-MS; Δ = 6.28 ppm for C_17_H_19_O_4_^−^), formed by the loss of CO_2_. This structure generates two main fragments in MS3, corresponding respectively to the loss of an isoprenyl from the geranyl chain (signal at *m*/*z* 217) and to different fragments (*m*/*z* 151 and 136) formed by the rupture of the same chain (Figure S3 and Fragmentation Scheme S3, Supplementary Material).

The MS2 spectrum of geranyl-PIB shows also signals at *m*/*z* 313 (*m*/*z* 313.1796 from HR-MS; Δ = 4.40 ppm for C_20_H_25_O_3_^−^), corresponding to the elimination of a molecule of water; 262 (*m*/*z* 262.1192 from HR-MS; Δ = 7.68 ppm for C_15_H_18_O_4_^−^), 233 (*m*/*z* 233.0803 from HR-MS; Δ = 7.28 ppm for C_13_H_13_O_4_^−^) and 194 (*m*/*z* 194.0577 from HR-MS; Δ = 4.37 ppm for C_10_H_10_O_4_^−^), corresponding to different breakages of the geranyl chain; and 207 (*m*/*z* 207.1744 from HR-MS; Δ = 5.11 ppm for C_14_H_23_O^−^), attributable to the entire geranyl chain (Fragmentation Scheme S3, Supplementary Material).

**Figure 5:**
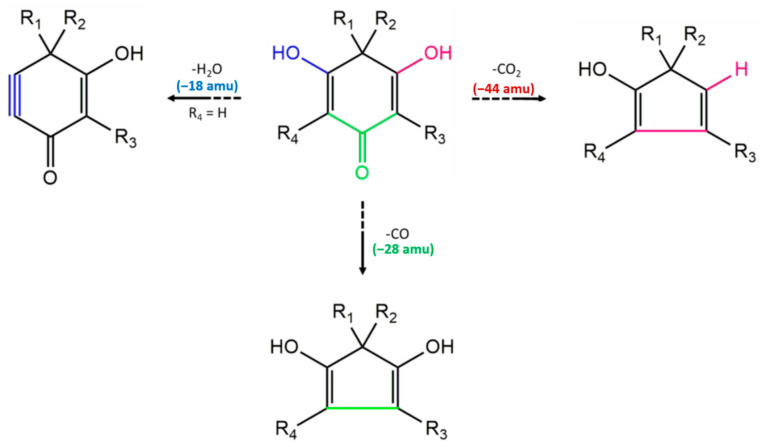
Characteristic proposed fragmentations for monomeric PIB derivatives. The “blue” path leads to the elimination of a molecule of water and to the formation of a triple bond. The “green” path leads to the elimination of a carbonyl group, with further rearrangement of the ring. The “purple” path leads to the elimination of CO_2_, followed by rearrangement of the ring.

As can be observed from the data shown above, polysubstituted monomeric PIB derivatives undergo characteristic fragmentations, detectable through typical mass losses that could be exploited for compound identification (Figure 5). Specifically, frequent losses of 44, 28 and 18 amu are observed, attributable respectively to the loss of a molecule of CO_2_, CO or water. The first two involve the breakage of the PIB ring, followed by rearrangement to a cyclopentadiene derivative, while the elimination of water leads to the formation of a triple bond (Figure 5). Similar fragmentation patterns have been already observed in flavonoids, where the fragments formed after the loss of CO and CO_2_ from pyrane and substituted benzene, respectively, are stabilized by a rearrangement to five-membered rings [16]. In the case of 3′3′me6′oxoPIB, we supposed that this intramolecular stabilization drives the loss of CO and CO_2_ from the ring, instead from the side chain. In fact, this latter would require an intermolecular rearrangement, and hence more energy.

**Figure 6:**
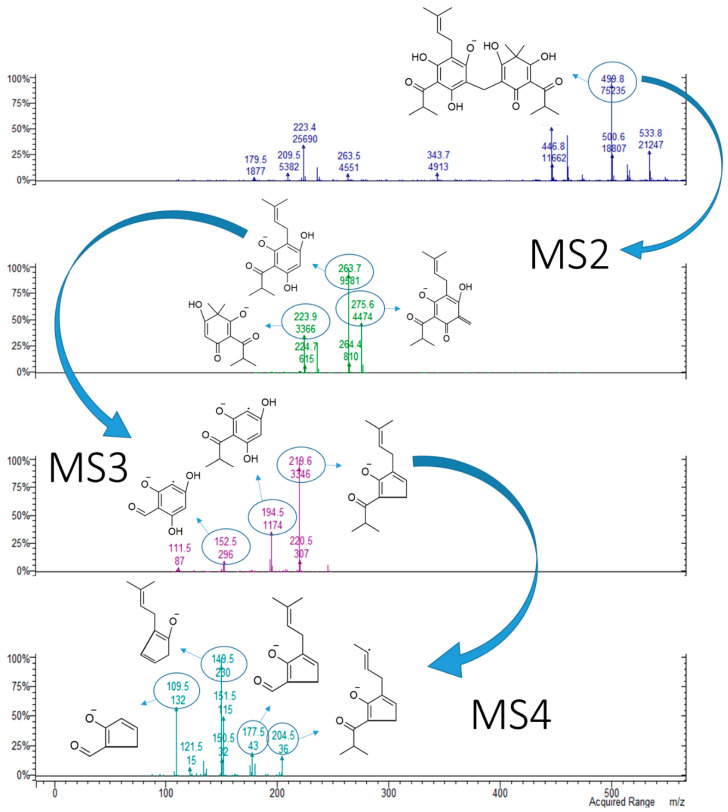
MS^n^ (*n* = 4) spectra of compound **9** (uliginosin A), [M-H]^−^ = 499. The structures of the fragments associated with the main peaks are reported.

Sarothralens A, C, D and G; saroaspidin A and saroaspidin B; and uliginosin A present the same core structure, characterized by the substituted phloroglucinol ring bonded to 3′3′me6′oxoPIB by a methylene bridge. Hence, their MS2 fragmentation spectra present common fragments, i.e., at *m*/*z* 235 (*m*/*z* 235.0960 from HR-MS; Δ = 3.61 ppm for C_13_H_15_O_4_^−^) and *m*/*z* 223, corresponding to the cyclohexa-2,5-dien-1-one moiety with or without the residue methylene group in position 6. As already observed and reported in the scheme in Figure 4, MS3 fragmentation of the structure at *m*/*z* 223 yields a base peak at *m*/*z* 179, due to the formal loss of CO_2_, which in turn yields the major fragment at *m*/*z* 109 at the MS4 level, corresponding to the loss of an isobutyl moiety. As a generic rule, the presence of both the signals at *m*/*z* 235 and 223 in the MS2 spectra of phloroglucinols from *H. japonicum* could be considered as indicative of dimeric derivatives, which can be further identified by the detection of peculiar fragments in the MS/MS spectra of the substituted phloroglucinol ring, as reported below. Exemplificative MS2–MS4 spectra of a dimeric phloroglucinol (uliginosin A) are reported in Figure 6. 

The MS2 spectrum of sarothralen A (*m*/*z* 567.2944 from HR-MS; Δ = 2.62 ppm for C_33_H_44_O_8_^−^) (Figure S4) shows several fragmentation pathways from different portions of the molecule (Fragmentation Scheme S4, Supplementary Material). The fragment at *m*/*z* 343 (*m*/*z* 343.1904 from HR-MS; Δ = 3.40 ppm for C_21_H_27_O_4_^−^) corresponds to the polysubstituted phloroglucinol portion bringing the residue of the methylene bridge in position 6, while that at *m*/*z* 331 (*m*/*z* 331.1901 from HR-MS; Δ = 4.48 ppm for C_20_H_27_O_4_^−^) corresponds to the same portion without the methylene residue. This latter fragment gives the structure at *m*/*z* 314 (*m*/*z* 314.1798 from HR-MS; Δ = 3.72 ppm for C_20_H_25_O_3_^−^) after the elimination of a molecule of water.

The MS2 spectrum of saroaspidin B (*m*/*z* 459.2005 from HR-MS; Δ = 4.38 ppm for C_25_H_31_O_8_^−^) presents a base peak ion at *m*/*z* 413 (*m*/*z* 413.1952 from HR-MS; Δ = 4.62 ppm for C_24_H_29_O_6_^−^), indicating the elimination of a molecule of water and a carbonyl group (-18 and -28 amu, respectively) from the cyclohexadienone ring (Figure S5), and a signal at *m*/*z* 388 (*m*/*z* 388.1512 from HR-MS; Δ = 4.37 ppm for C_21_H_24_O_7_^−^), corresponding to a fragment generated by the loss of the isobutyl moiety from the cyclohexadienone (Fragmentation Scheme S5, Supplementary Material).

The fragment at *m*/*z* 413 undergoes several fragmentation pathways in MS3, leading to the formation of the base peak at *m*/*z* 343 and the fragments at *m*/*z* 395, 370, 299 and 177. The base peak at *m*/*z* 343 indicates the rupture of the rearranged isobutyl moiety from the parent compound, and after the loss of CO_2_, generates the fragment at *m*/*z* 299. The fragment at *m*/*z* 395 is generated by the elimination of a molecule of water, and that at *m*/*z* 370 is generated by sequential loss of a CO and a methyl radical. Finally, the structure at *m*/*z* 177 corresponds with the 1,1,5-trimethyl-4-oxo-1,4,5,6-tetrahydropentalen-2-olate portion of the structure at *m*/*z* 413, after the rupture of the methylene bridge linking the two core rings. 

The MS2 spectrum of sarothralen G (*m*/*z* 601.2780 from HR-MS; Δ = 4.76 ppm for C_36_H_41_O_8_^−^) shows fragments generated from three different fragmentation pathways (Figure S6). The rupture of the methylene bridge linking the phloroglucinol ring with 3′3′me6′oxoPIB leads to fragments at *m*/*z* 377 (*m*/*z* 377.1743 from HR-MS; Δ = 4.22 ppm for C_24_H_25_O_4_^−^) and 365 (*m*/*z* 365.1743 from HR-MS; Δ = 4.35 ppm for C_23_H_25_O_4_^−^), together with those at 235 and 223. The first two correspond to the phloroglucinol portion with and without the methylene residue, respectively. Other two fragments, at *m*/*z* 389 (*m*/*z* 389.1229 from HR-MS; Δ = 3.54 ppm for C_20_H_21_O_8_^−^) and 347 (*m*/*z* 347.0750 from HR-MS; Δ = 6.72 ppm for C_17_H_15_O_8_^−^), indicate the loss of the geranyl chain from the phloroglucinol ring, followed by the rupture of the aromatic ring from the ketone on the same portion of the structure (fragment at *m*/*z* 389), and the rupture of the ketones in both phloroglucinol and 3′3′me6′oxoPIB rings (fragment at *m*/*z* 347) (Fragmentation Scheme S6, Supplementary Material).

Saroaspidin A (*m*/*z* 445.1845 from HR-MS; Δ = 5.48 ppm for C_24_H_29_O_8_^−^) shows a simple MS2 spectrum (Figure S7), in which the signals could be assigned to the fragments formed after the rupture of the methylene bridge (Fragmentation Scheme S7, Supplementary Material). Fragments at *m*/*z* 235 and 221 (*m*/*z* 221.0807 from HR-MS; Δ = 5.74 ppm for C_12_H_13_O_4_^−^) keep the residue bonded, while those at *m*/*z* 223 and 209 (*m*/*z* 209.0808 from HR-MS; Δ = 5.77 ppm for C_11_H_13_O_4_^−^) do not. 

In the MS2 spectrum of uliginosin A (*m*/*z* 499.2314 from HR-MS; Δ = 4.88 ppm for C_28_H_35_O_8_^−^), the fragments at *m*/*z* 275 (*m*/*z* 275.1275 from HR-MS; Δ = 5.40 ppm for C_16_H_19_O_4_^−^) and 235 are those that keep the methylene residue bonded, while those at *m*/*z* and 263 (*m*/*z* 263.1277 from HR-MS; Δ = 4.83 ppm for C_15_H_19_O_4_^−^) and 223 do not (Figure 5 and Fragmentation Scheme S8, Supplementary Material). In the MS3 spectrum, fragmentation of the structure at *m*/*z* 263 gives the fragment at *m*/*z* 194 by the loss of 2-methylbut-2-ene, which in turn gives the fragment at *m*/*z* 152 after the loss of C_3_H_6_ from the ketone in the side chain of the aromatic ring. The base peak of MS3, corresponding to the fragment at *m*/*z* 219, is formed by the loss of a molecule of CO_2_ from the parent compound. This latter fragment could generate several signals in MS4 by different breakages of the 2-methylbut-2-ene moiety or the ketone in the side chain. The loss of a methyl radical gives the fragment at *m*/*z* 204, and the complete loss of the alkyl chain leads to the signal at *m*/*z* 151. The latter could undergo the elimination of C_3_H_6_ (−42 amu) from the ketone in the side chain, leading to the signal at *m*/*z* 109. The loss of C_3_H_6_ from the structure at *m*/*z* 219 gives the fragment at *m*/*z* 177, which, after the sequential loss of CO and a methyl radical, gives the fragments at *m*/*z* 149 and 134, respectively.

Sarothralens C and D show the same MW (*m*/*z* 583.2888 from HR-MS; Δ = 4.54 ppm for C_33_H_43_O_9_^−^) and the same fragmentation pathways, being isomers (Fragmentation Scheme S9, Supplementary Material). MS2 spectra (Figure S8) show signals at *m*/*z* 347 (*m*/*z* 347.1845 from HR-MS; Δ = 5.80 ppm for C_20_H_27_O_5_^−^), 235, 223 and 359 (*m*/*z* 359.1852 from HR-MS; Δ = 3.54 ppm for C_21_H_27_O_5_^−^) formed by the rupture of the methylene bridge. The structure at *m*/*z* 359 can undergo the loss of isobutene from the 4-methylpentene bonded to the tetrahydropyran, giving the signal at *m*/*z* 303 (*m*/*z* 303.1222 from HR-MS; Δ = 5.60 ppm for C_17_H_19_O_5_^−^). However, the spectra of the two isomers do not contain distinctive signals that allow their differentiation; hence, using only MS data it is not feasible for distinguishing between the two.

## 3. Materials and Methods

### 3.1. Plant Collection and Identification

Plant specimens were collected in June 2010 in Budhathum, Dhading District, Nepal (N 28° 05’ 29.8’’, E 84° 50’ 57.4’’) at an altitude of 1000 m. Plant specimens were tagged in the field during collection with appropriate field note. The collected plants were dried and mounted on herbarium sheets. The identification of plant specimens was performed by a team of botanists headed by S. Rajbhandary, using relevant literature [17,18,19,20]. A voucher specimen was deposited at the Tribhuvan University Central Herbarium (TUCH), Kathmandu, Nepal, under the representative code: EK0420.

### 3.2. Extraction of Plant Material

Extraction was performed using the aerial parts of the plant, as already reported in [11]. Briefly, 50 g of dried and ground plant material was added to 250 mL of MeOH and extracted in an ultrasound bath (FALC, Italy) for 2 h at room temperature. After centrifugation at 4000 rpm for 10 min, the powder was recovered and subjected to a further extraction for 30 min, using an additional 250 mL of MeOH. After filtration, the solvent was dried under vacuum at 40 °C to constant weight, and after weighting, the residue was stored at −20 °C until analysis. The whole extraction procedure was performed in triplicate. The mean percentage yield of the crude HJME obtained was 19 ± 1.5%.

### 3.3. HPLC-MS^n^ and UPLC-QTOF Separation and Characterization of Phloroglucinols

Phloroglucinol derivatives in HJME were characterized using an integrated HPLC-MS^n^ and UPLC-QTOF approach, as already reported in [11]. For both HPLC-MS^n^ and UPLC-QTOF analyses, the samples were prepared dissolving 50 mg of dried extract in 5 mL of methanol and sonicated for 20 min. The samples were then centrifuged at 13,000 rpm and used for analysis. HPLC-MS^n^ characterization was conducted using an Agilent 1260 binary pump coupled to a Varian MS 500 mass detector (3D ion trap). Electrospray (ESI) was employed as the ion source, operating in negative (ESI(−)) ion mode. An Agilent Eclipse plus C18 column (2.1 × 150 mm, 3.5 µm) was used as the stationary phase, while the mobile phase was composed of acetonitrile (A) and 0.1% formic acid in water (B). The gradient was as follows: 0 min, 10% A; 20 min, 54% A; 23 min, 100% A; isocratic up to 35 min. Re-equilibration time was 8 min. Flow rate was 200 µL min^−1^. ESI parameters were as follows: needle voltage, 4500 V; capillary voltage, 70 V; RF loading, 100%; nebulizing gas pressure, 20 psi (nitrogen); drying gas pressure, 15 psi; drying gas temperature, 350 °C. Mass range was 50–2000 Da. Fragmentation patterns of eluted compounds were obtained using the turbo detection data scanning (TDDS^®^) function of the instrument, setting *n* = 4 levels of fragmentation.

Accurate *m*/*z* values (ppm < 10) were obtained using a Waters Acquity H-Class UPLC system coupled to a Waters Xevo G2 QTOF MS detector, operating in ESI(−) mode. The chromatographic conditions were kept as previously described. MS parameters were as follows: sampling cone voltage, 40 V; source offset, 80 V; capillary voltage, 3500 V; nebulizer gas (N_2_) flow rate, 800 L/h; desolvation temperature, 450 °C. The mass accuracy and reproducibility were maintained by infusing lockmass ([M−H]^−^ = 554.2620 *m*/*z*) thorough Lockspray at a flow rate of 20 μL/min. Centroided data were collected in the mass range 50−1200 Da, and *m*/*z* values were automatically corrected during acquisition using lockmass. Accurate *m*/*z* values of fragments were acquired while performing a parallel MS/MS experiment, keeping the collision energy constant at 30 V.

## 4. Conclusions

In this work, we reported the characterization of the multi-stage fragmentation pathways of eleven phloroglucinols extracted from Nepalese *H. japonicum* by means of LC-MS^n^ and LC-QTOF. In a previous work on the same plant species, we tentatively identified the phloroglucinols in a methanolic extract obtained from the aerial parts of *H. japonicum*, although the in-depth characterization of the fragmentation pathways and the identification of the main fragments generated from each constituent were missing. Exhaustive MS structural information about phloroglucinols from *H. japonicum* is hence reported here for the first time.

Low-resolution ion trap MS allowed us to observe characteristic fragmentation patterns for the classes of compounds studied, such as the loss of −44, −28 and −18 amu, attributable to CO_2_, CO and water from 3′3′me6′oxoPIB, respectively, but also significant differences in the MS^n^ spectra of the various phloroglucinols, especially those with dimeric structures. On the other hand, high-resolution QTOF analysis gave information about the accurate *m*/*z* value of each compound and about the MS2 fragments, allowing one to calculate their molecular formulas and support the data from the low-resolution MS. 

Overall, our results represent the first exhaustive mass spectrometric data about phloroglucinols from Nepalese *H. japonicum*, and they could be a useful starting point for those laboratories that will focus on the characterization and isolation of these compounds from natural sources.

## Supplementary Materials

The following are available online. Figures S1–S8: MS^n^ spectra of 2-acetyl-3,5-dihydroxy-1-geranoxy-6-methyl-4-(2-methyl)butyryl-benzene, 1′3′pren45′me4′oxoPIB, geranyl PIB, sarothralen A, saroaspidin B, sarothralen G, saroaspidin A, and sarothralens C and D, respectively. Fragmentation Schemes S1–S9: 2-acetyl-3,5-dihydroxy-1-geranoxy-6-methyl-4-(2-methyl)butyryl-benzene, 1′3′pren45′me4′oxoPIB, geranyl PIB, sarothralen A, saroaspidin B, sarothralen G, saroaspidin A, uliginosin A, and sarothralens C and D, respectively.
