# Supplementary material for: An Integrated LC-ESI-MSn and High Resolution LC-ESI-QTOF Approach for the Identification of Phloroglucinols from Nepalese Hypericum japonicum
Source: Molecules. 2020 Dec 15;25(24):5937. doi: 10.3390/molecules25245937 (PMC7765257; doi:10.3390/molecules25245937)
Supplement: Supplementary file 1 [file molecules-25-05937-s001.pdf]

An integrated LC-ESI-MS^n and High Resolution LC-ESI-QTOF approach for the identification of phloroglucinols from Nepalese *Hypericum japonicum*

Gregorio Peron [a*], Deepak Raj Pant [b], Shyam Sharan Shrestha [b], Sangeeta Rajbhandary [b], Stefano Dall'Acqua [a*]

Affiliations

[a] Department of Pharmaceutical and Pharmacological Sciences, University of Padova, Via Marzolo 5, 35131 Padova, Italy. **Gregorio Peron**: e-mail: gregorio.peron@unive.it; ORCID number: 0000-0002-6007-6184. **Stefano Dall'Acqua**: e-mail: stefano.dallacqua@unipd.it; ORCID number: 0000-0001-8264-6953.

[b] Central Department of Botany, Tribhuvan University, 44600 Kirtipur, Kathmandu, Nepal. **Sangeeta Rajbhandary**: e-mail: s.rajbhandary@cdbtu.edu.np; **Deepak Raj Pant**: drpant_agbot@yahoo.com; **Shyam Sharan Shestha**: e-mail: shyamsharan999@gmail.com.

*Correspondence: Stefano Dall'Acqua: stefano.dallacqua@unipd.it; Gregorio Peron: gregorio.peron@unive.it.

SUPPLEMENTARY MATERIAL

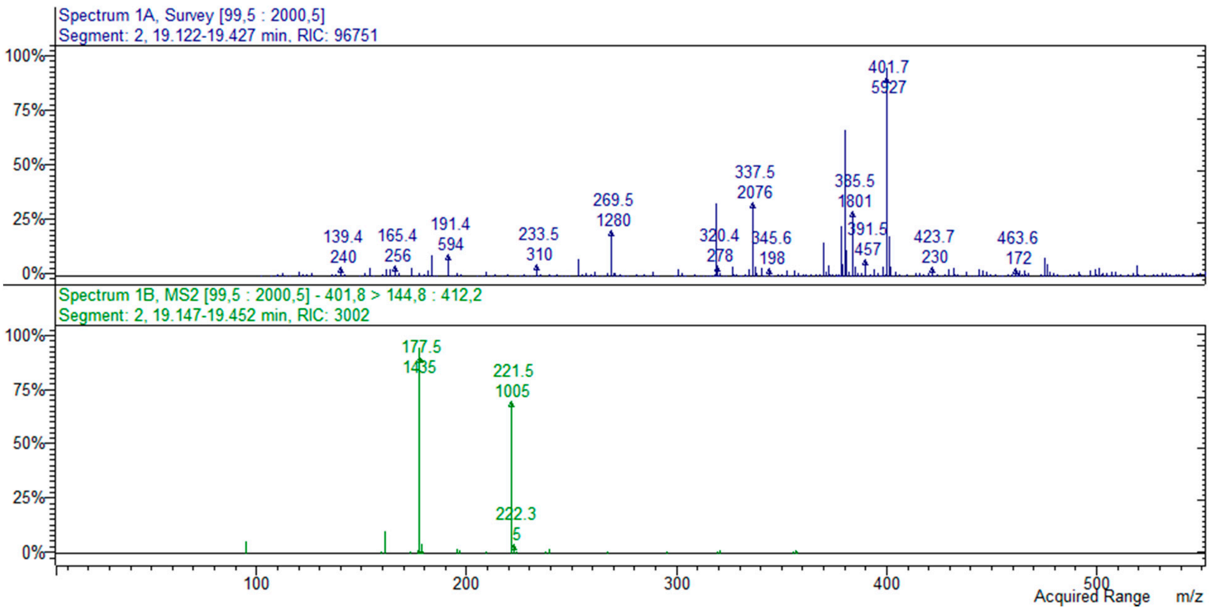

Figure S1. MS/MS spectrum of 2-acetyl-3,5-dihydroxy-1-geranoxy-6-methyl-4-(2-methyl)butyryl-benzene, $[M-H]^-$ = 401.

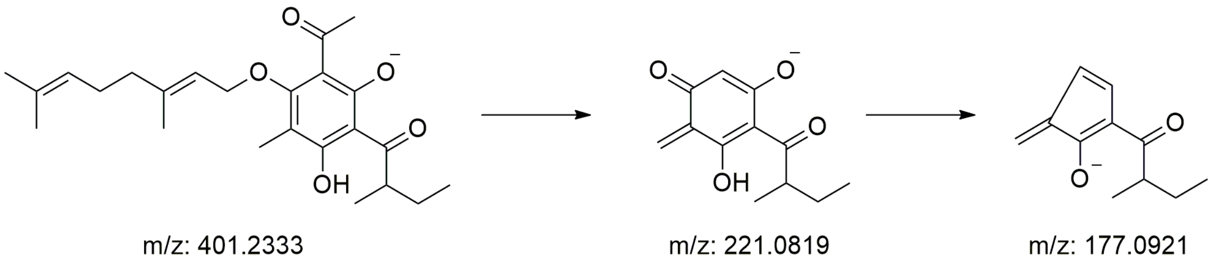

Fragmentation Scheme 1. 2-acetyl-3,5-dihydroxy-1-geranoxy-6-methyl-4-(2-methyl)butyryl-benzene, $[M-H]^-$ = 401.

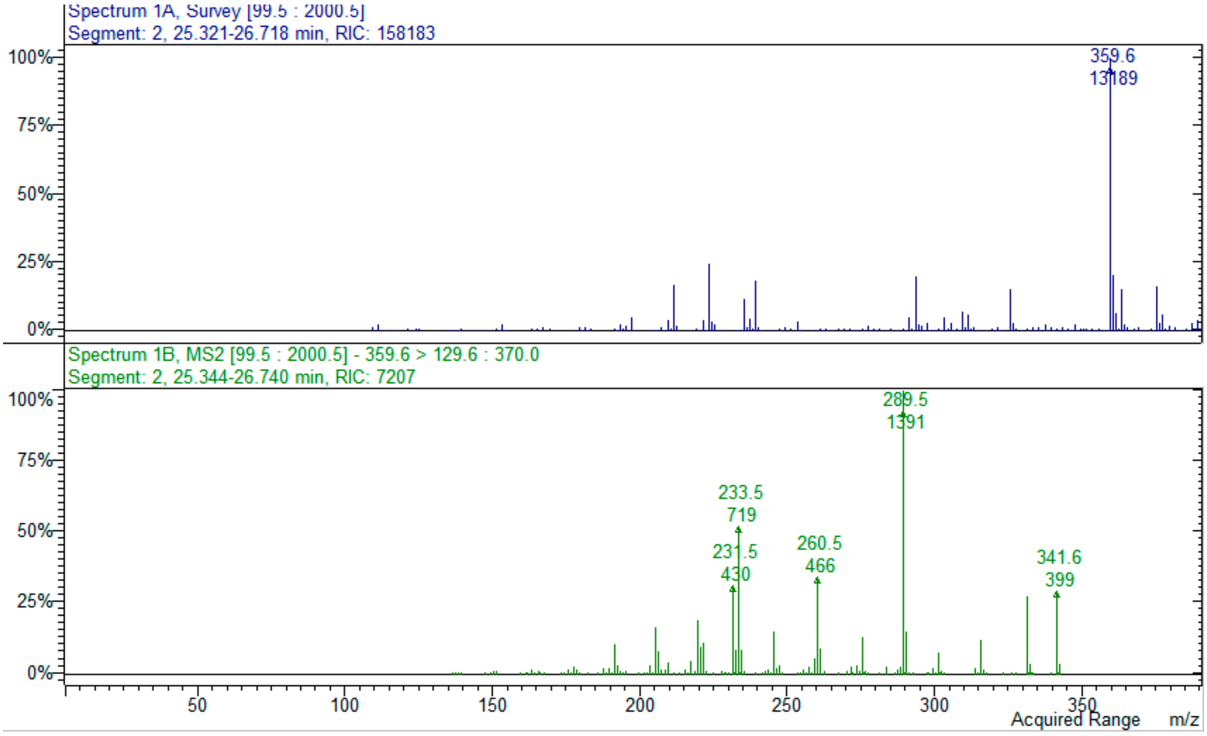

Figure S2. MS/MS spectrum of 1′3′pren45′me4′oxoPIB, [M-H]$^-$ = 359.

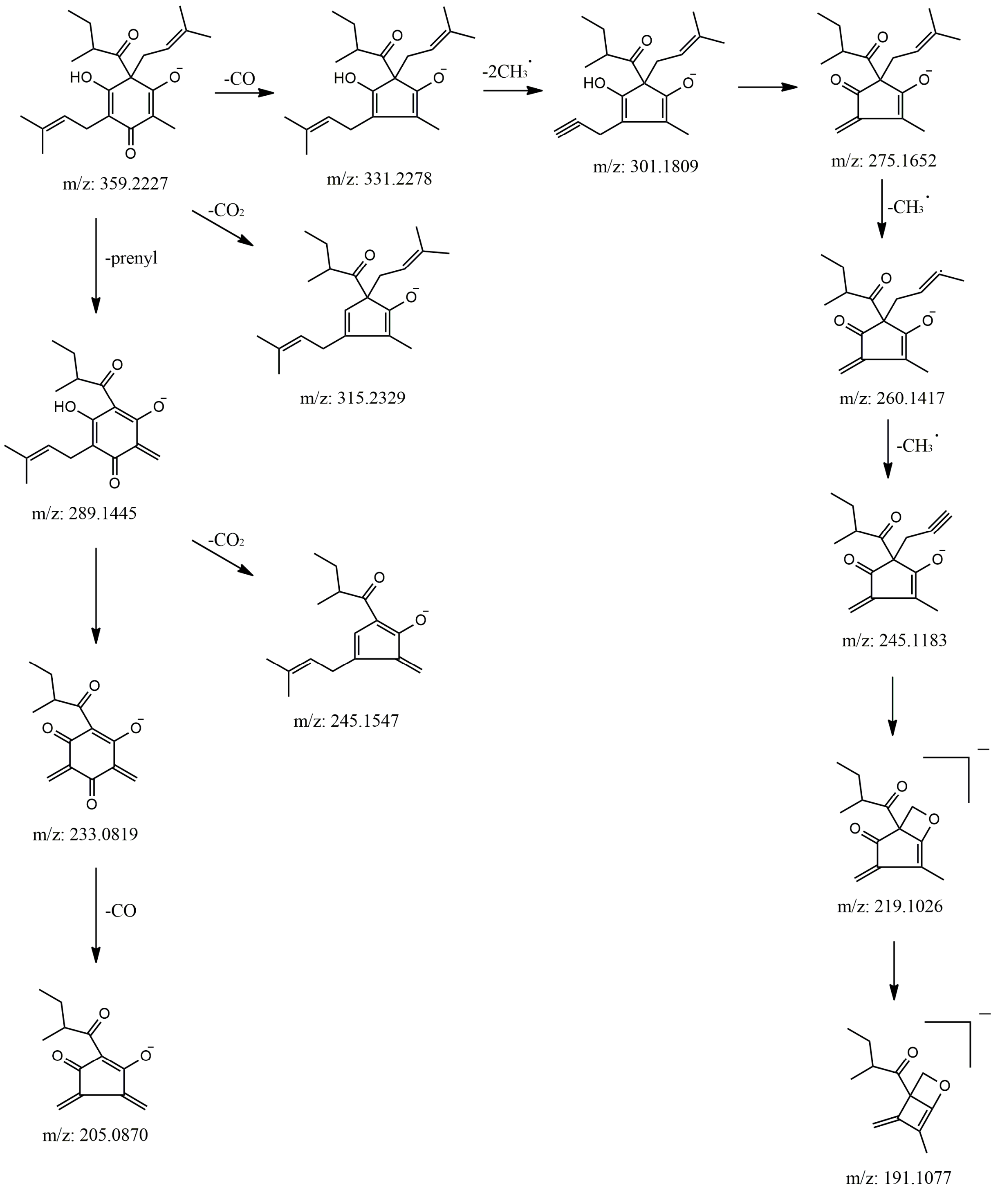

Fragmentation Scheme 2. 1′3′pren45′me4′oxoPIB, $[M\text{-}H]^-$ = 359.

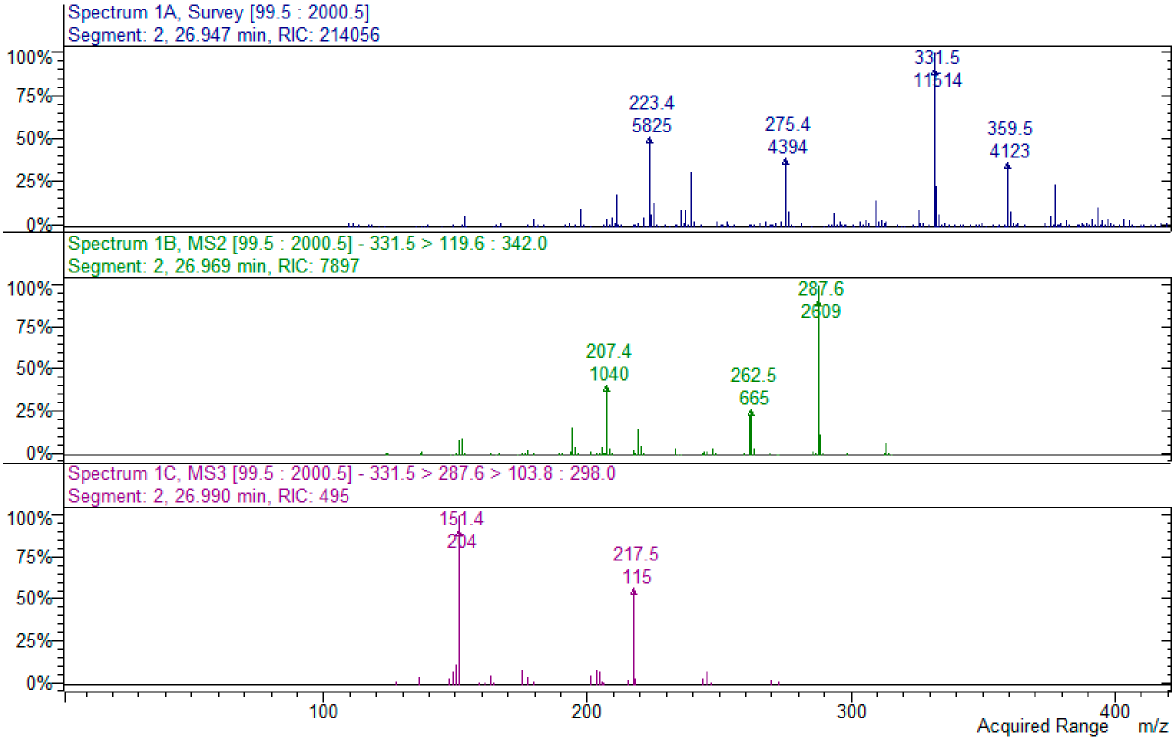

Figure S3. MS^n spectra (n=3) of geranyl phlorisobutyrophenone, $[M-H]^-$ = 331.

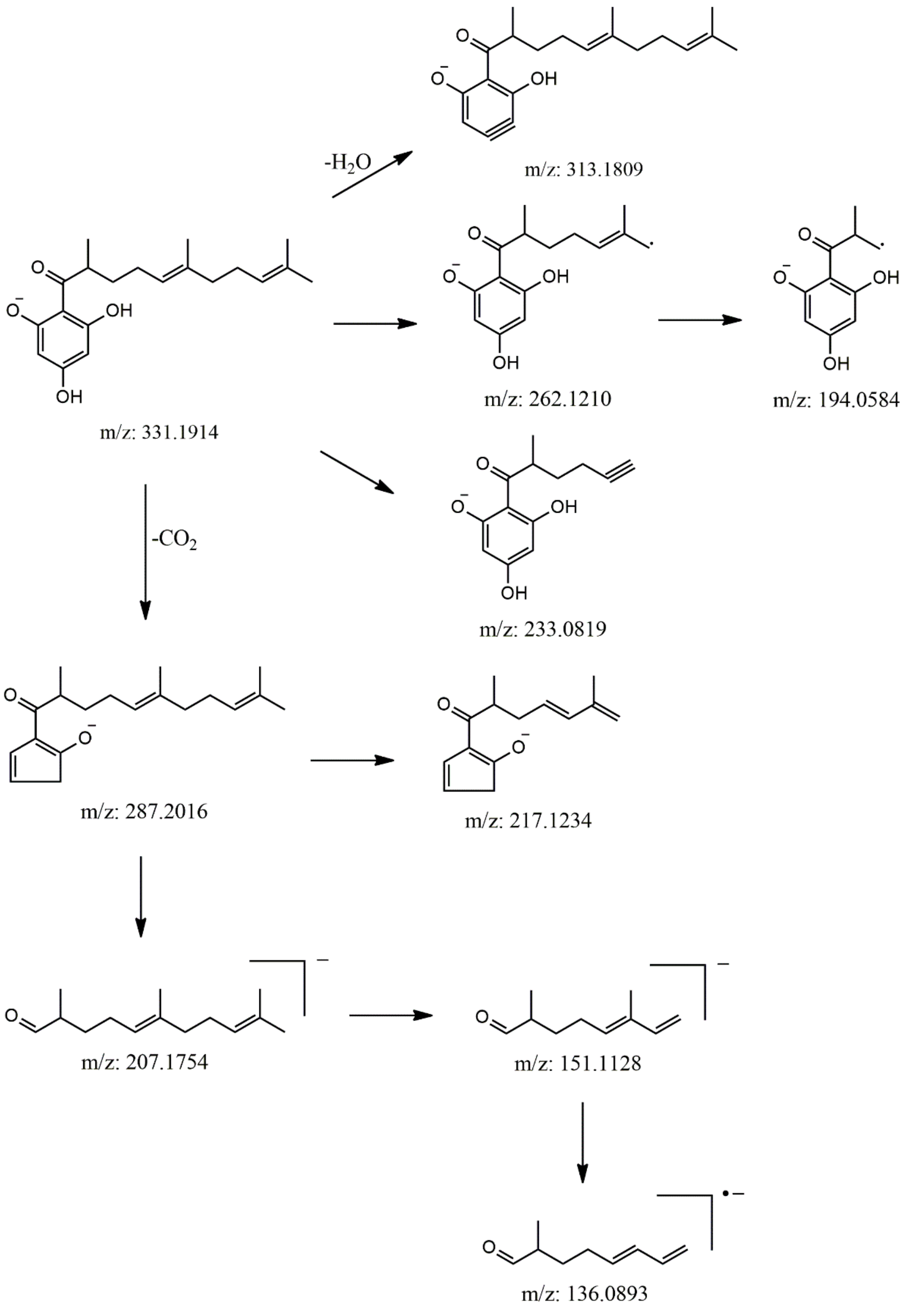

Fragmentation Scheme 3. Geranyl phlorisobutyrophenone, $[M-H]^-$ = 331.

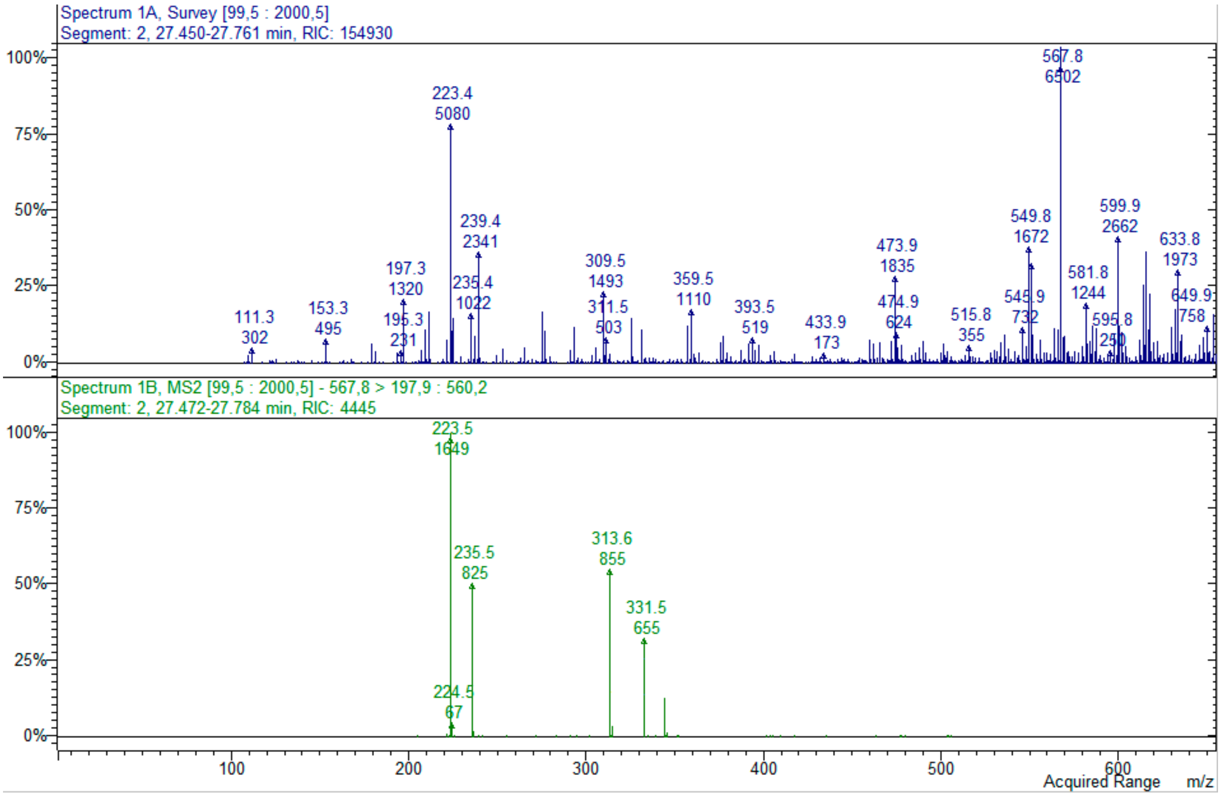

Figure S4. MS/MS spectrum of sarothralen A, $[M-H]^-$ = 567.

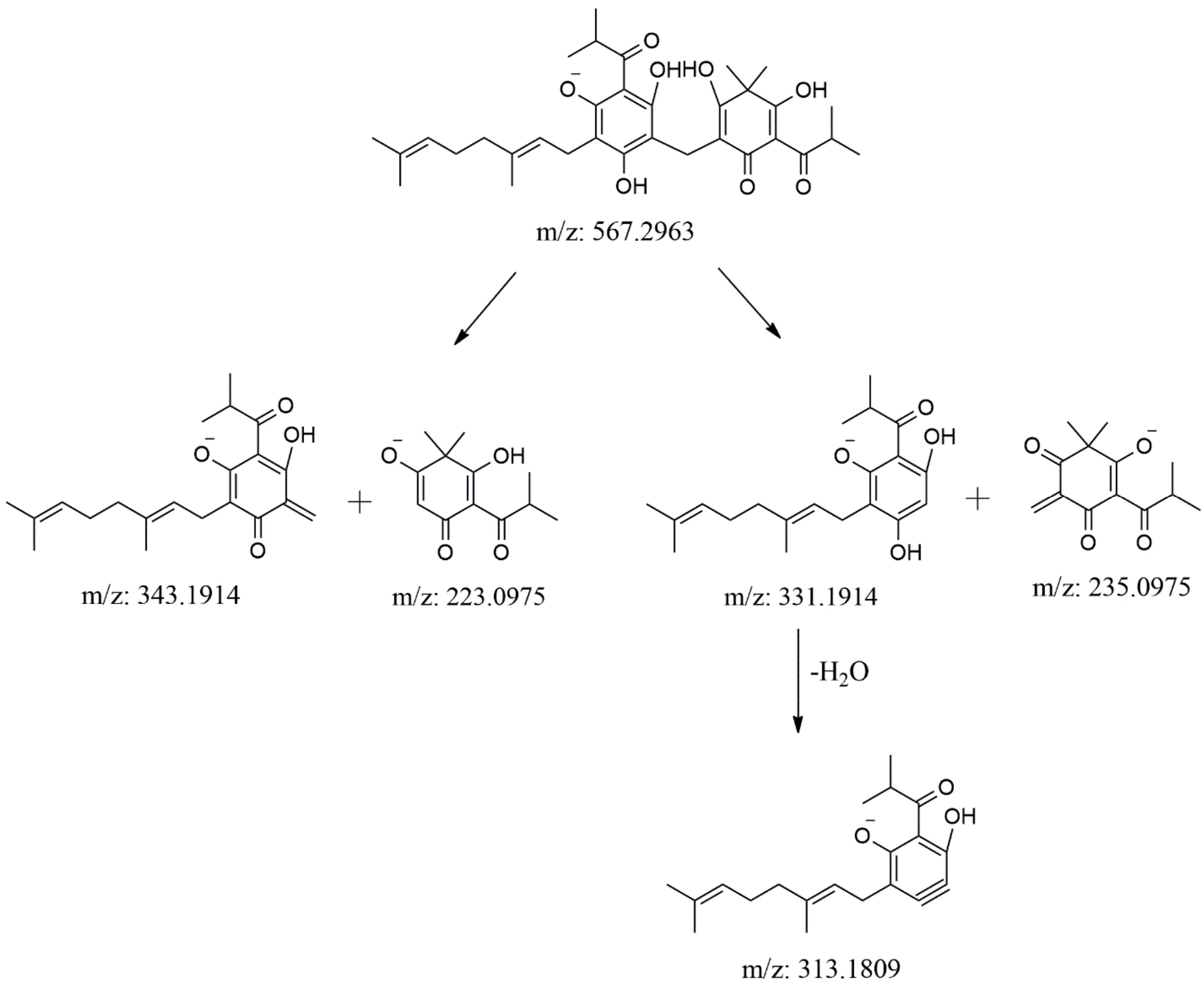

Fragmentation Scheme 4. Sarothralen A, $[M-H]^-$ = 567.

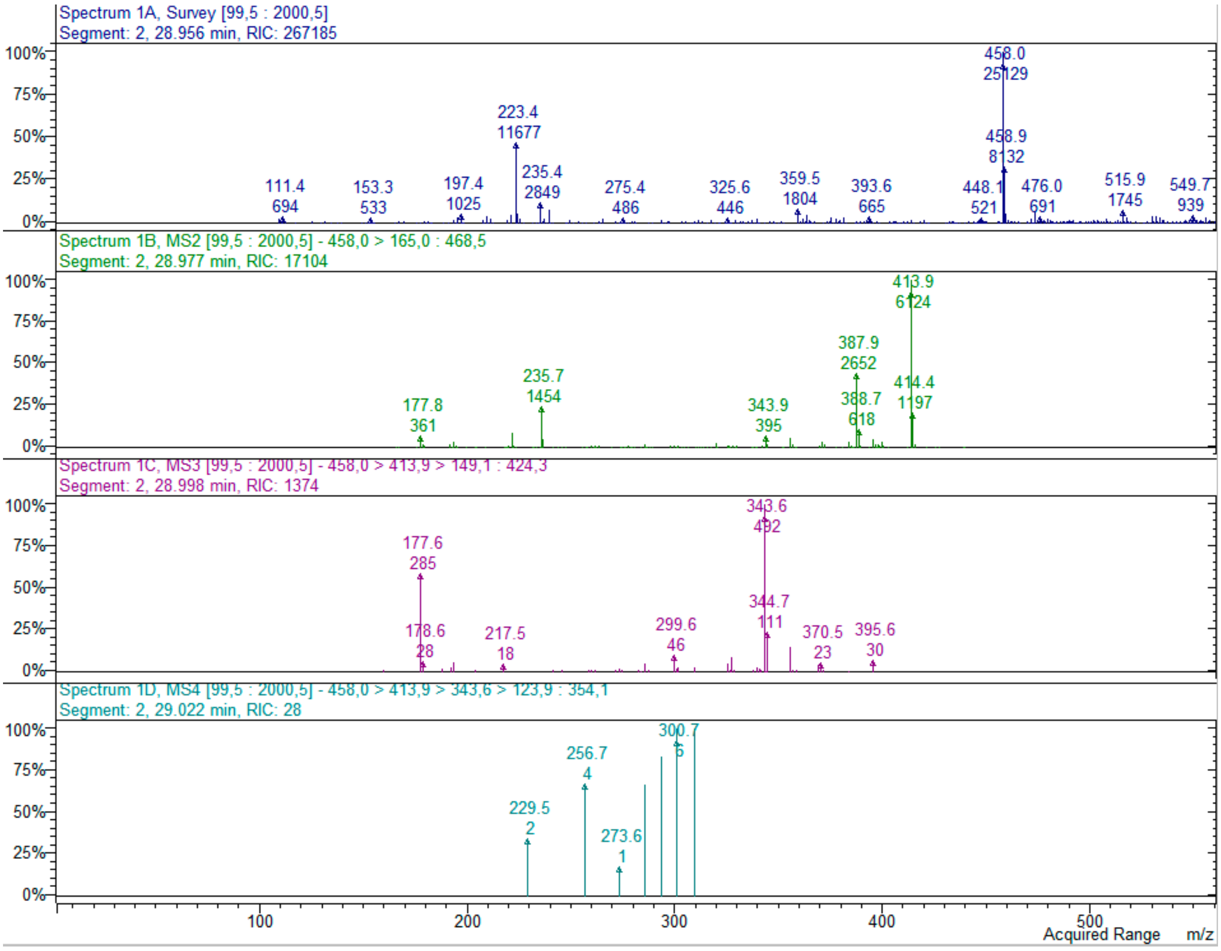

Figure S5. MS^n spectra (n = 4) of saroaspidin B, $[M-H]^-$ = 459.

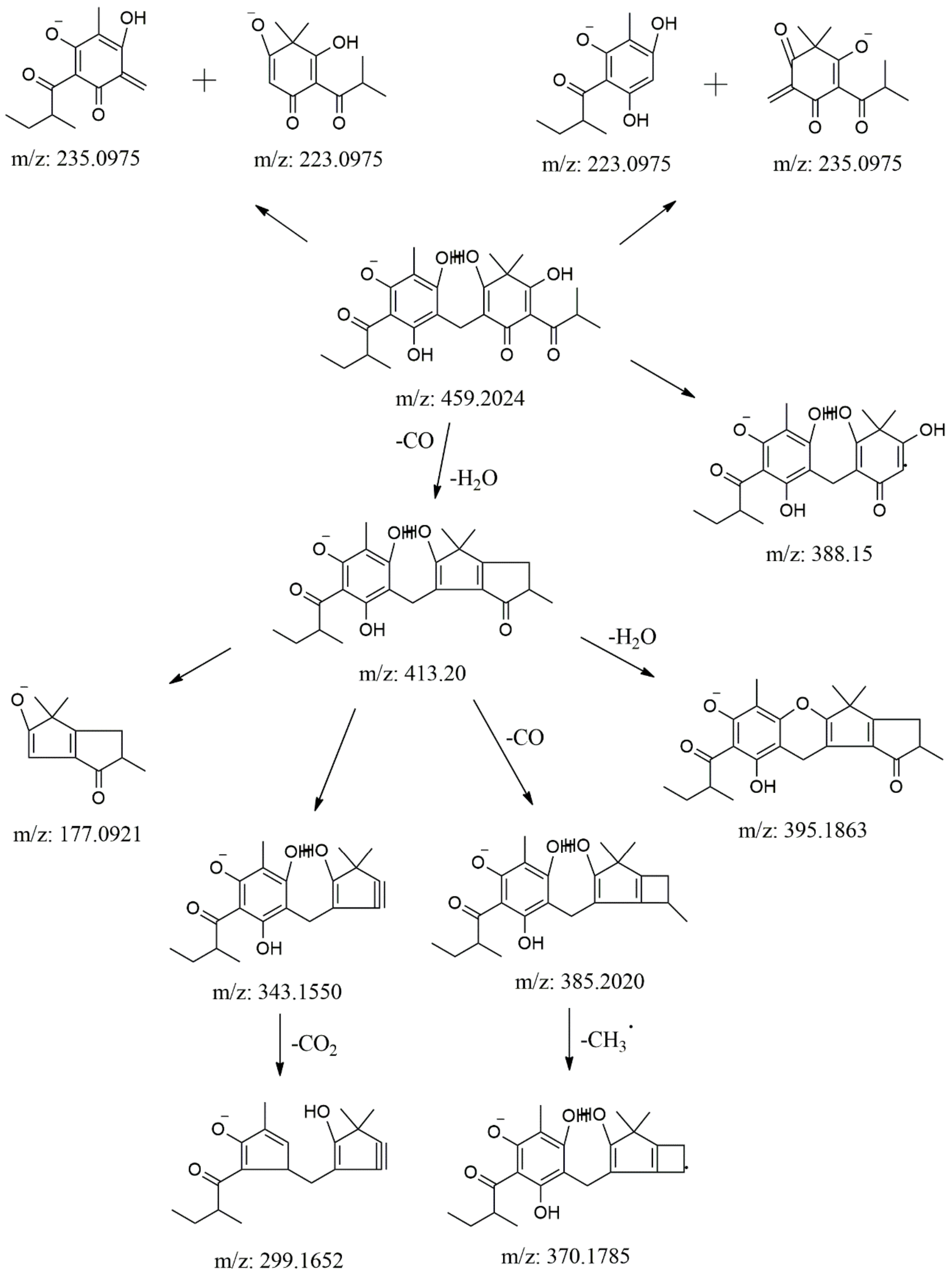

Fragmentation Scheme 5. Saroaspidin B, $[M\text{-}H]^- = 459$.

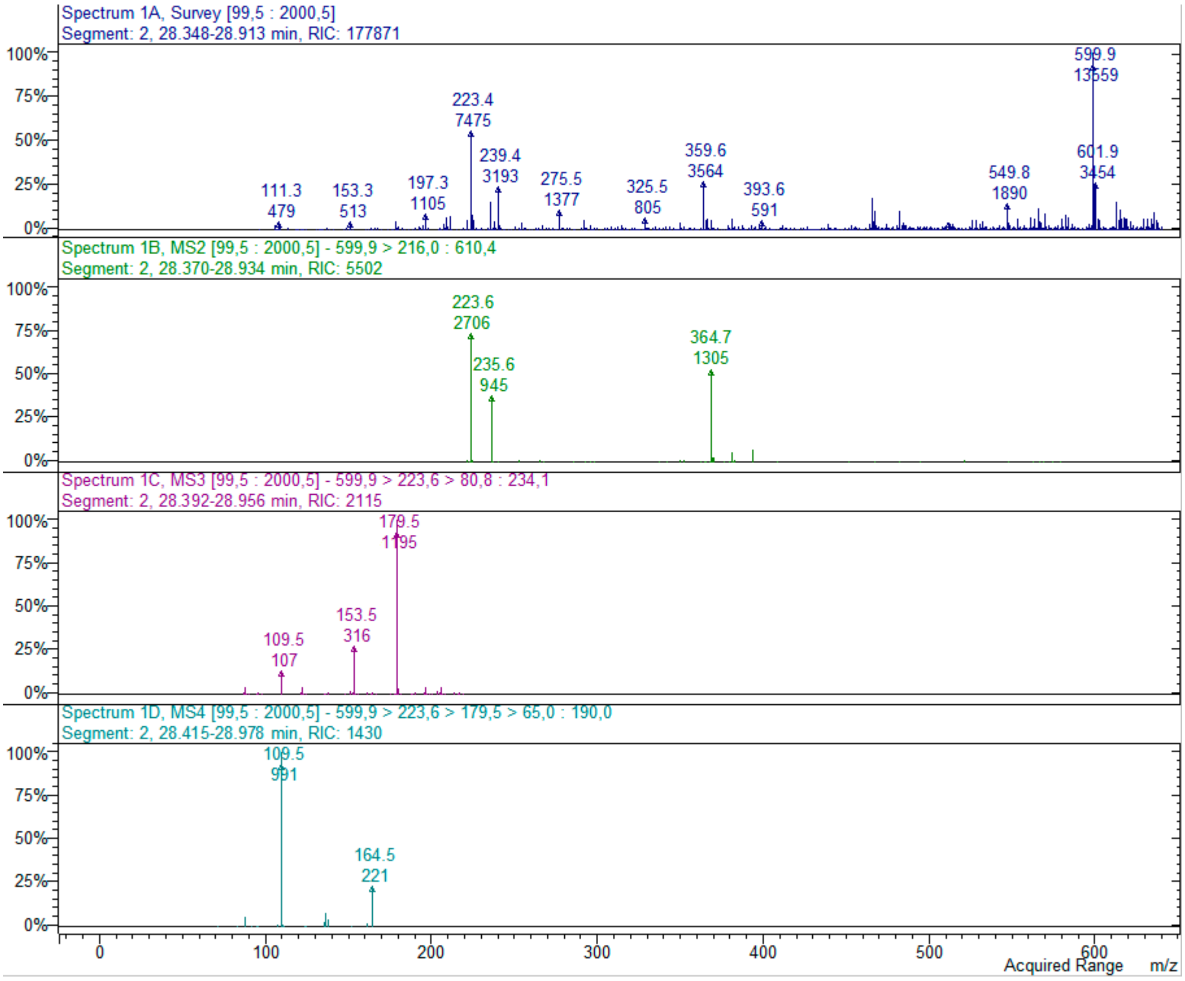

Figure S6. MS^n spectra (n = 4) of sarothralen G, $[M-H]^-$ = 601.

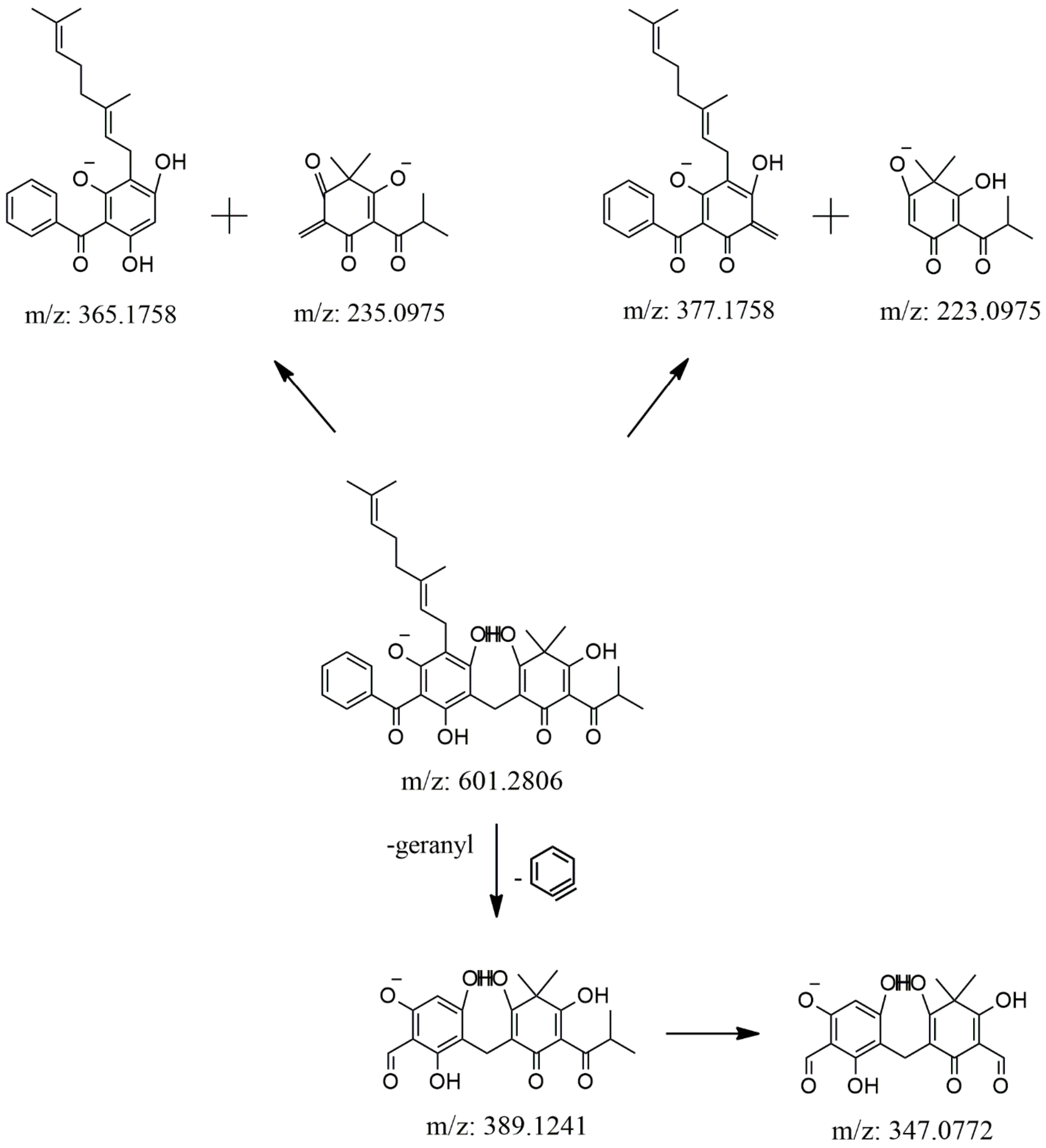

Fragmentation Scheme 6. Sarothralen G, $[M-H]^-$ = 601.

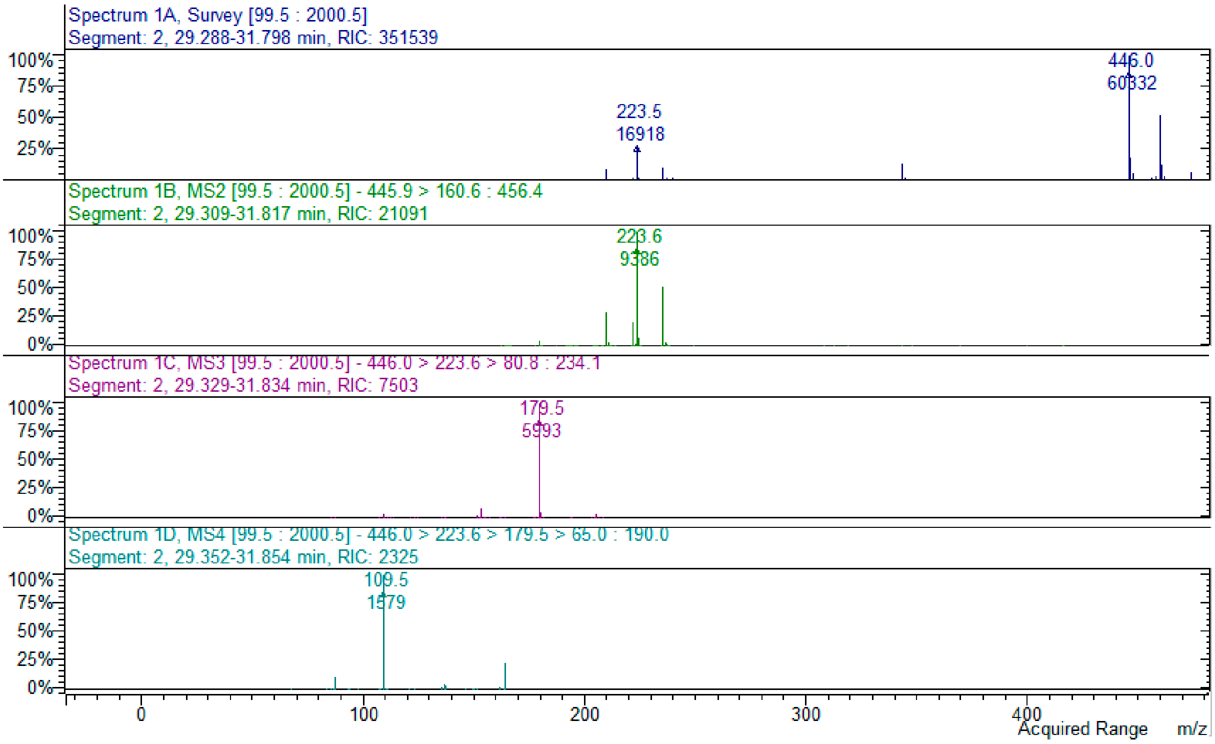

Figure S7. MS/MS spectrum of saroaspidin A, $[M-H]^-$ = 445.

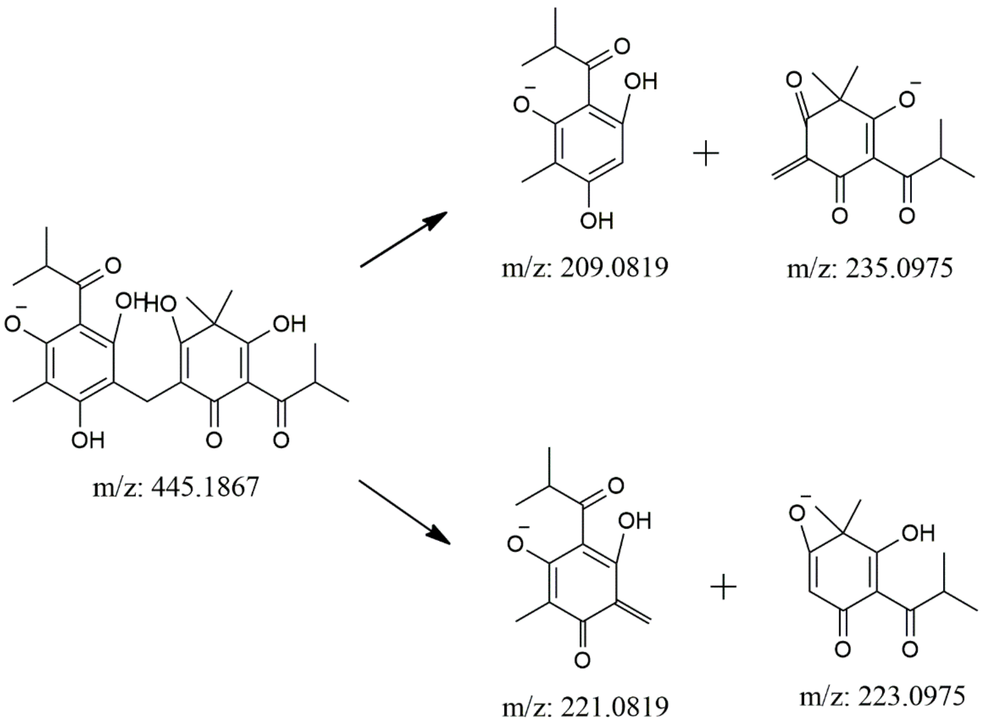

Fragmentation Scheme 7. Saroaspidin A, $[M-H]^-$ = 445.

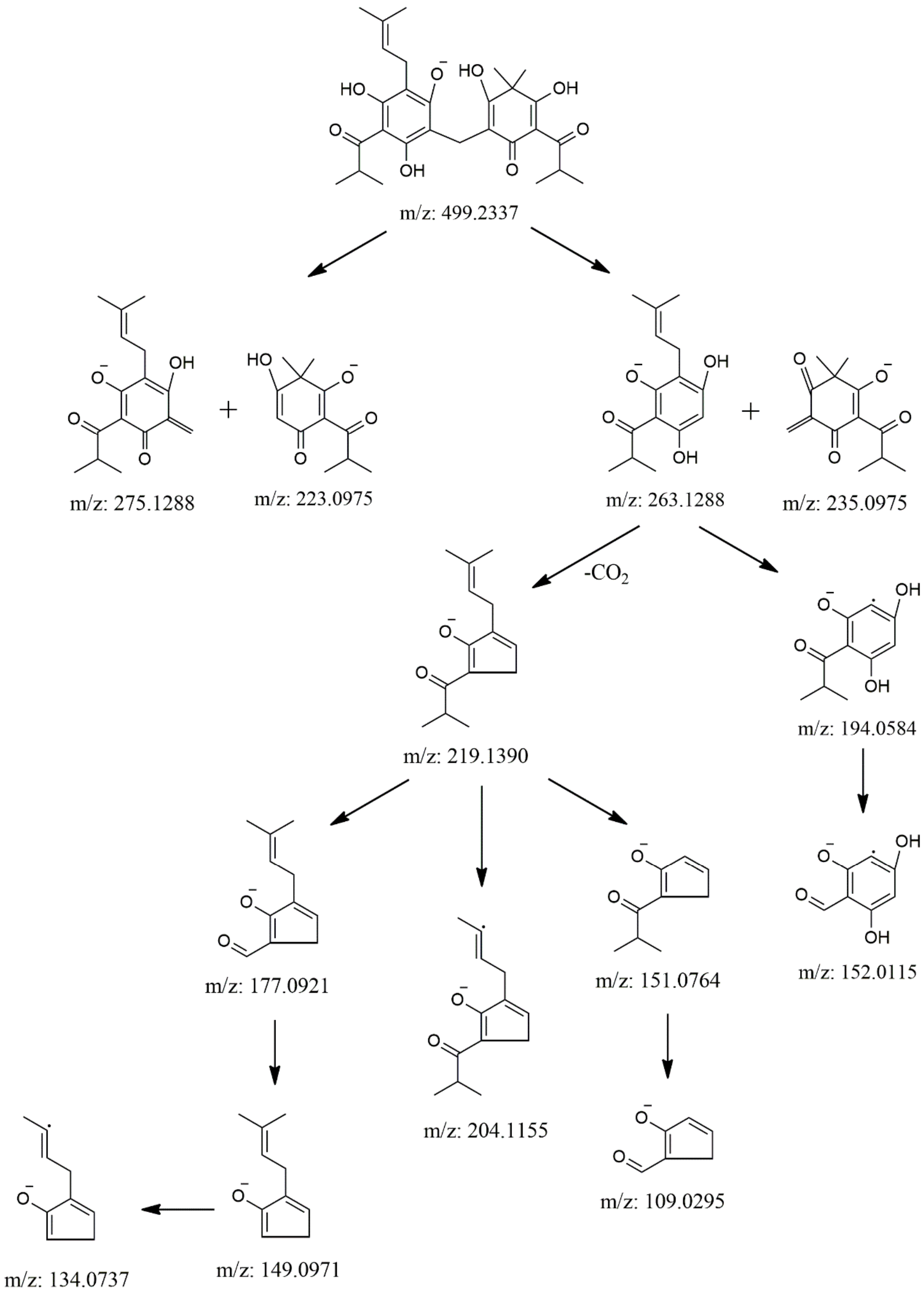

Fragmentation Scheme 8. Uliginosin A, $[M-H]^-$ = 499.

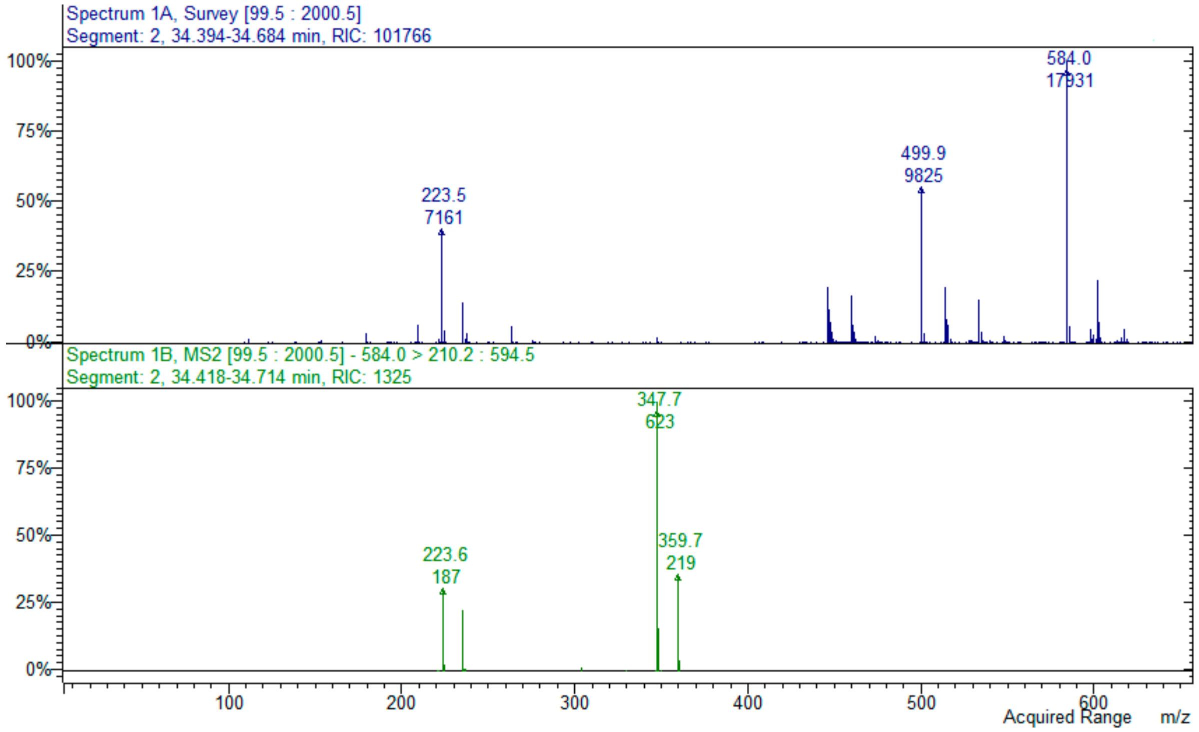

Figure S8. MS/MS spectrum of sarothralens C and D, $[M-H]^-$ = 583.

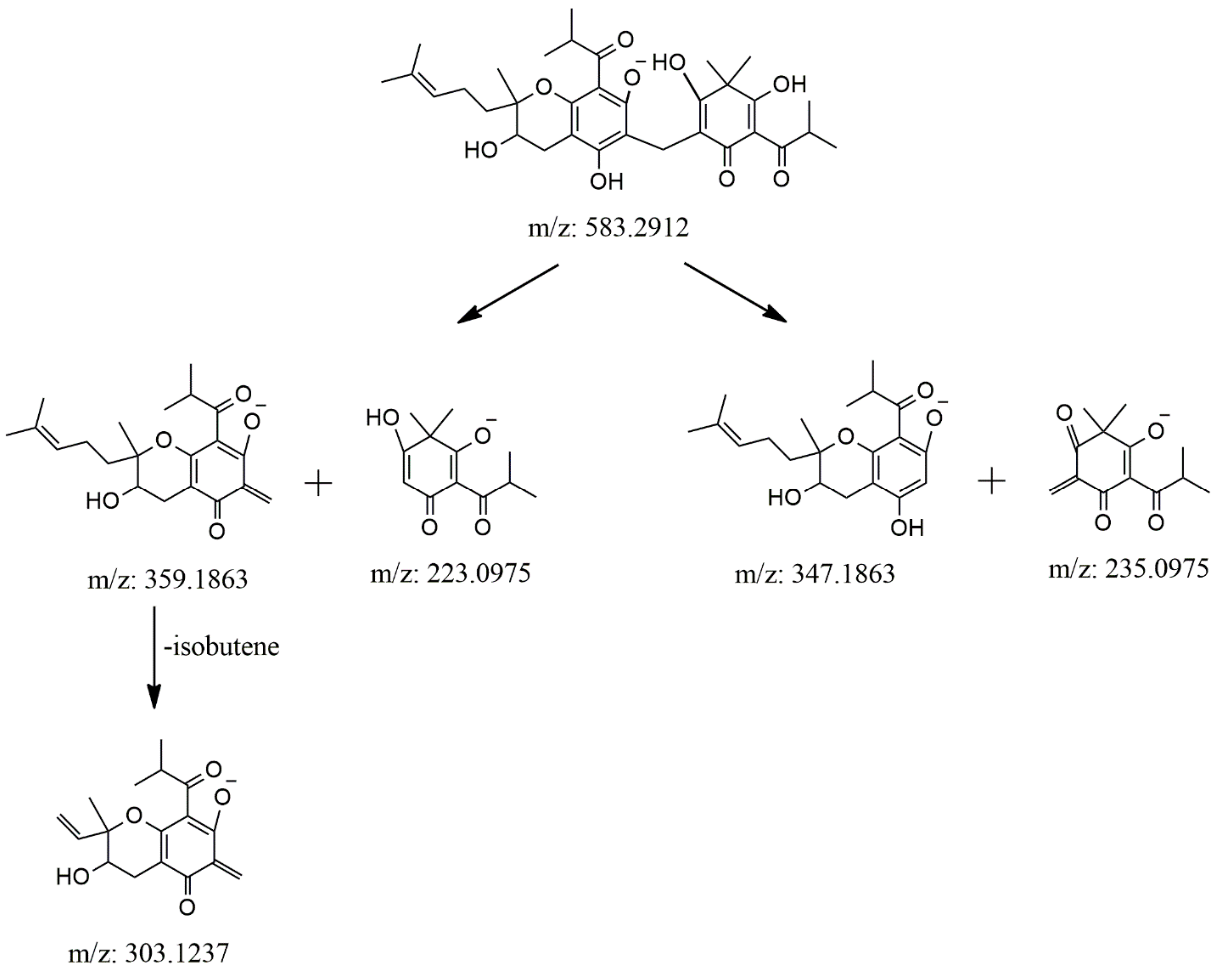

Fragmentation Scheme 9. Sarothralens C and D, [M-H]$^-$ = 583.
